# Sub-Acute Rheumatism

**Published:** 1871-01

**Authors:** 


### SUB-ACUTE RHEUMATISM.

Clinic by PROF. DAVIS. Reported by S.

MERCY HOSPITAL, *Dec. 16, 1870.*

This patient came here a week ago to-morrow; had been sick nearly a week previous. On admission, was laboring under a well-marked sub-acute form of rheumatism, complaining more of his lower extremities; knees and ankles swollen; quite painful and helpless. The inflammation then moved to the upper extremities, involving the elbow and wrist, first of one side and then the other, producing considerable swelling and pain, which was aggravated on movement.

On admission, finding with the above symptoms considerable fever and a harsh cough, he was put upon a solution of

℞ Muriat ammonia, ʒ iii.

Tart. ant. et. pot., 2 grs.

Sulph. morph., 3 grs.

Syrup liquorice, ℥ iv.

Given every four hours, alternately with a powder, consisting of Dover's powder 6 grs., bi-carb. soda 6 grs., hydrarg. chl. M. 1 gr., it being intended that the Dover's powder should be sufficient to prevent the calomel and soda from acting as a cathartic, while the two latter would exert an alkaline and alterative effect. I supposed, also, that the morphine contained in the muriate of ammonia mixture would aid the Dover's powder in allaying the irritation of the bronchial tubes, and, in conjunction with the tart. antimony and muriate ammonia, would be likely to give relief to the soreness in the chest. Continued this treatment four days, during which time the cough improved, the pain in the chest was relieved, and the pain and swelling in the extremities subsided. Thinking the calomel had been taken as long as was judicious, I directed it to be left off, and to give the Dover's powder and soda alone alternately with the ammonia mixture, but yesterday a pretty active disturbance of the bowels had supervened, with decided diarrhœa, pains in the abdomen, and sickness at the stomach. It was not easy to attribute this to the action of the medicine, as I had already left out the calomel, which was about the only thing that would operate as physic, the muriate ammonia mixture generally constipating the bowels, unless combined with some laxative to maintain regularity.

I also noticed at this time a decided development of jaundice indicated by a distinct yellowish hue of the skin and conjunctiva. I attribute the diarrhœa to some irritation affecting the mucous membrane lining the hepatic duct, as well as the mucous membrane of the intestine. Ordered the ammonia mixture to be continued, and in the place of the powder substituted another, consisting of Bismuth subnit. 6 grs., soda bicarb. 6 grs., and morphia sulphas ¼ gr.

To-day the bowels are more quiet, the color of the skin much improved.

Last night and to-day he has a little rheumatism in his wrist and hands; I would direct that the ammonia mixture be now restricted to a single dose morning and evening, and give the powders every three hours, so as to get more of the influence of the soda and Bismuth upon the system, and less of the ammonia mixture, which is now not so much needed.

If the bowels continue to be quiet and the yellow hue of the skin disappears, he might have a tablespoonful of the following solution every four hours, so as to get a more efficient action of the kidneys. The morphine in the powders would prevent the pot. bitart. from having too much action upon the bowels. The solution consists of the following:

℞ Potas. bitart., ʒ ij.

Potas. acetas, ʒ iv.

Aqua dist., ℥ viij.

